# Programmed cell death (PCD): an essential process of cereal seed development and germination

**DOI:** 10.3389/fpls.2014.00366

**Published:** 2014-07-28

**Authors:** Fernando Domínguez, Francisco J. Cejudo

**Affiliations:** Instituto de Bioquímica Vegetal y Fotosíntesis, Universidad de Sevilla – Consejo Superior de Investigaciones CientíficasSevilla, Spain

**Keywords:** cereal, development, germination, plant, programmed cell death, seed

## Abstract

The life cycle of cereal seeds can be divided into two phases, development and germination, separated by a quiescent period. Seed development and germination require the growth and differentiation of new tissues, but also the ordered disappearance of cells, which takes place by a process of programmed cell death (PCD). For this reason, cereal seeds have become excellent model systems for the study of developmental PCD in plants. At early stages of seed development, maternal tissues such as the nucellus, the pericarp, and the nucellar projections undergo a progressive degeneration by PCD, which allows the remobilization of their cellular contents for nourishing new filial tissues such as the embryo and the endosperm. At a later stage, during seed maturation, the endosperm undergoes PCD, but these cells remain intact in the mature grain and their contents will not be remobilized until germination. Thus, the only tissues that remain alive when seed development is completed are the embryo axis, the scutellum and the aleurone layer. In germinating seeds, both the scutellum and the aleurone layer play essential roles in producing the hydrolytic enzymes for the mobilization of the storage compounds of the starchy endosperm, which serve to support early seedling growth. Once this function is completed, scutellum and aleurone cells undergo PCD; their contents being used to support the growth of the germinated embryo. PCD occurs with tightly controlled spatial-temporal patterns allowing coordinated fluxes of nutrients between the different seed tissues. In this review, we will summarize the current knowledge of the tissues undergoing PCD in developing and germinating cereal seeds, focussing on the biochemical features of the process. The effect of hormones and redox regulation on PCD control will be discussed.

## INTRODUCTION

**FIGURE 1:**
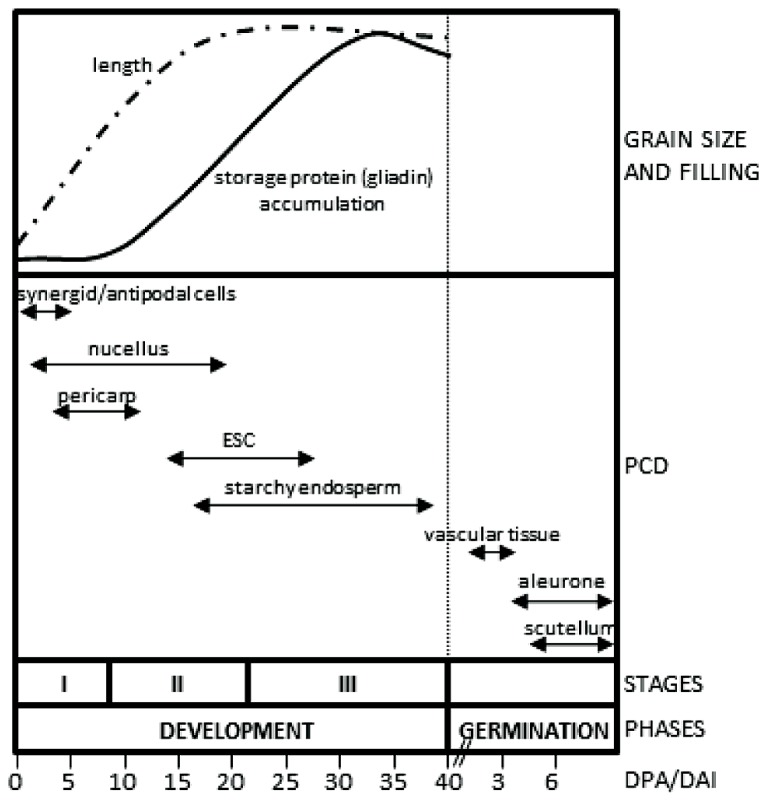
**Tissues undergoing PCD in developing and germinating cereal seeds.** The upper panel represents the increase in seed length and storage accumulation taking as models the wheat and barley seeds. The lower panel indicates the periods of seed development in which the tissues undergo PCD. Stages of development: I, early development; II, differentiation; III, maturation. ESC, embryo-surrounding cells; DPA, days post anthesis (development); DAI, days after imbibition (germination).

Two phases may be distinguished in the life cycle of the cereal seeds, development and germination. Seed development is initiated by the fertilization events and culminates with the formation of a mature seed, which has a low content of water and remains in a quiescent status. Upon imbibition, the quiescent seed initiates the phase of germination in which the reserves stored in the starchy endosperm are remobilized to support the initial stages of seedling growth. The phase of development may be subdivided in three stages (**Figure 1**): (I), early development, which includes double fertilization, syncytium formation and endosperm cellularization; (II), differentiation, which comprises the formation of the different cell types of the seed (embryo-surrounding cells, transfer cells, starchy endosperm and aleurone), endoreduplication and accumulation of storage reserves in the endosperm; and (III), maturation, which includes desiccation and dormancy (Sabelli and Larkins, 2009). At the morphological level, early seed development is characterized by a large increase in seed size, which reaches about 80% of its final length at this stage (**Figure 1**; Bosnes et al., 1992; Domínguez and Cejudo, 1996). Seed length increases predominantly due to the elongation of the pericarp cells in the longitudinal direction; this growth making room to accommodate the growing endosperm (Radchuk et al., 2011). Prior to the stage of storage accumulation in the growing endosperm, a remobilization of nutrients from maternal tissues takes place, which involves the participation of a complex set of hydrolytic enzymes, including proteases (Domínguez and Cejudo, 1996) and starch-degrading enzymes (Radchuk et al., 2009). Following germination, a large process of remobilization of the storage compounds of the starchy endosperm occurs. This process, which supports early seedling growth until the new plant becomes autonomous, requires the synthesis and secretion of hydrolytic enzymes initially by the scutellum epithelium cells. Then, the aleurone cells, which surround the starchy endosperm, become activated and secrete a large amount of hydrolases (Fincher, 1989), in a process regulated by gibberellins synthesized by the scutellum and then released into the starchy endosperm (Appleford and Lenton, 1997).

Although the formation of a new seed, as well as the generation of a new plant upon germination, requires the generation and differentiation of a large number of cells, the ordered disappearance of cells is also of high relevance. Cell degeneration takes place by programmed cell death (PCD), which may be considered as an ordered process of selective removal of cells. There are several reasons that make PCD an important process for the successful completion of seed development and germination. Both seed development and germination rely on a continuous remobilization of nutrients, which is supported by cell degeneration. This is the case of antipodal, nucellar and pericarp cells, which degenerate at early stages of seed development (Domínguez et al., 2001; An and You, 2004; Radchuk et al., 2011). The starchy endosperm undergoes PCD during the final stages of seed development; however, this tissue remains intact after cell death in the mature seed and the reserves stored therein will be mobilized only after germination. PCD also has the important function of creating new structures, such as nucellar projection cells at middle stages of seed development (Domínguez et al., 2001), and the vascular tissue of the scutellum, the differentiation of which is completed immediately after imbibition (Domínguez et al., 2002). Finally, PCD of pericarp cells is associated with the enlargement of the seed, facilitating the growth of the endosperm (Radchuk et al., 2011) and the formation of the seed cuticle, which has a protective function.

In summary, PCD is an essential process for the successful completion of cereal seed development and germination and, thus, cereal seeds have become an important model system for the study of cell death in plants. Based on morphological characteristics, two categories of plant PCD were recently proposed: vacuolar PCD, which includes autophagy, and necrosis, in conjunction with some forms of PCD that present mixed features and are not clearly ascribed to these categories (van Doorn et al., 2011). Most of the tissues that undergo PCD in cereal seeds show features of vacuolar PCD in agreement with the notion that cell death is part of developmental programs that characterize the formation and germination of the seed. In this review we will summarize the present knowledge of the tissues undergoing PCD in developing and germinating cereal seeds, emphasizing their morphological and biochemical characteristics. In addition, we will focus on the function of the process of PCD for the successful completion of these developmental programs.

## PCD OF MATERNAL TISSUES IS AN IMPORTANT PROCESS OF CEREAL SEED DEVELOPMENT

In monocots, fertilization is a double event that results in a diploid embryo and a triploid endosperm (Olsen, 2004). The formation of the seed involves the generation, growth and differentiation of new tissues; however, the ordered disappearance of cells plays as well an important function in this complex developmental process. In particular, several maternal tissues undergo PCD to help the formation of the seed; among them, it is worth mentioning the deaths of cells of the embryo sac, the nucellus, the pericarp, and the nucellar projections, which take place sequentially, as outlined in **Figure 1**. It follows below a brief description of how these cells undergo PCD.

### SYNERGID AND ANTIPODAL CELLS

The embryo sac is composed of an egg cell, which is accompanied by two synergid cells at one pole and three antipodal cells at the opposite pole. Synergid cells are the first to undergo PCD at this initial stage of seed development (**Figure 1**). This death process occurs shortly before the pollen tube discharges and seems to be important to guide the growth of the pollen tube (An and You, 2004). In *Arabidopsis*, it was shown that the signaling cascade leading to the death of one of the two synergids is initiated by the contact with the pollen tube (Sandaklie-Nikolova et al., 2007). Synergids control sperm delivery through the *FERONIA* signaling pathway to initiate and modulate their distinct calcium signatures in response to calcium dynamics and growth behavior of the pollen tube (Ngo et al., 2014). PCD of antipodal cells occurs later, at 2–3 days post-anthesis (DPA), and contributes to the development of the adjacent free-nuclear endosperm. Nuclear materials from the dying antipodal cells support the nuclear divisions in the growing coenocyte (Engell, 1994; An and You, 2004).

### NUCELLUS

**FIGURE 2:**
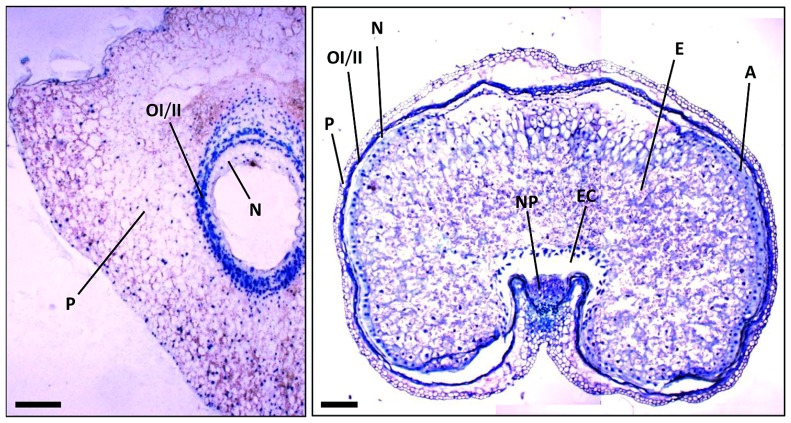
**Sections of wheat seeds at 3 DPA (left) and 16 DPA (right) stained with toluidine blue.** Note the different tissues and how they change between these stages of seed development. P, pericarp; OI, outer integument; II, inner integument; N, nucellus; NP, nucellar projections; A, aleurone; E, starchy endosperm; EC, endosperm cavity. Bars, 100 μm.

**FIGURE 3:**
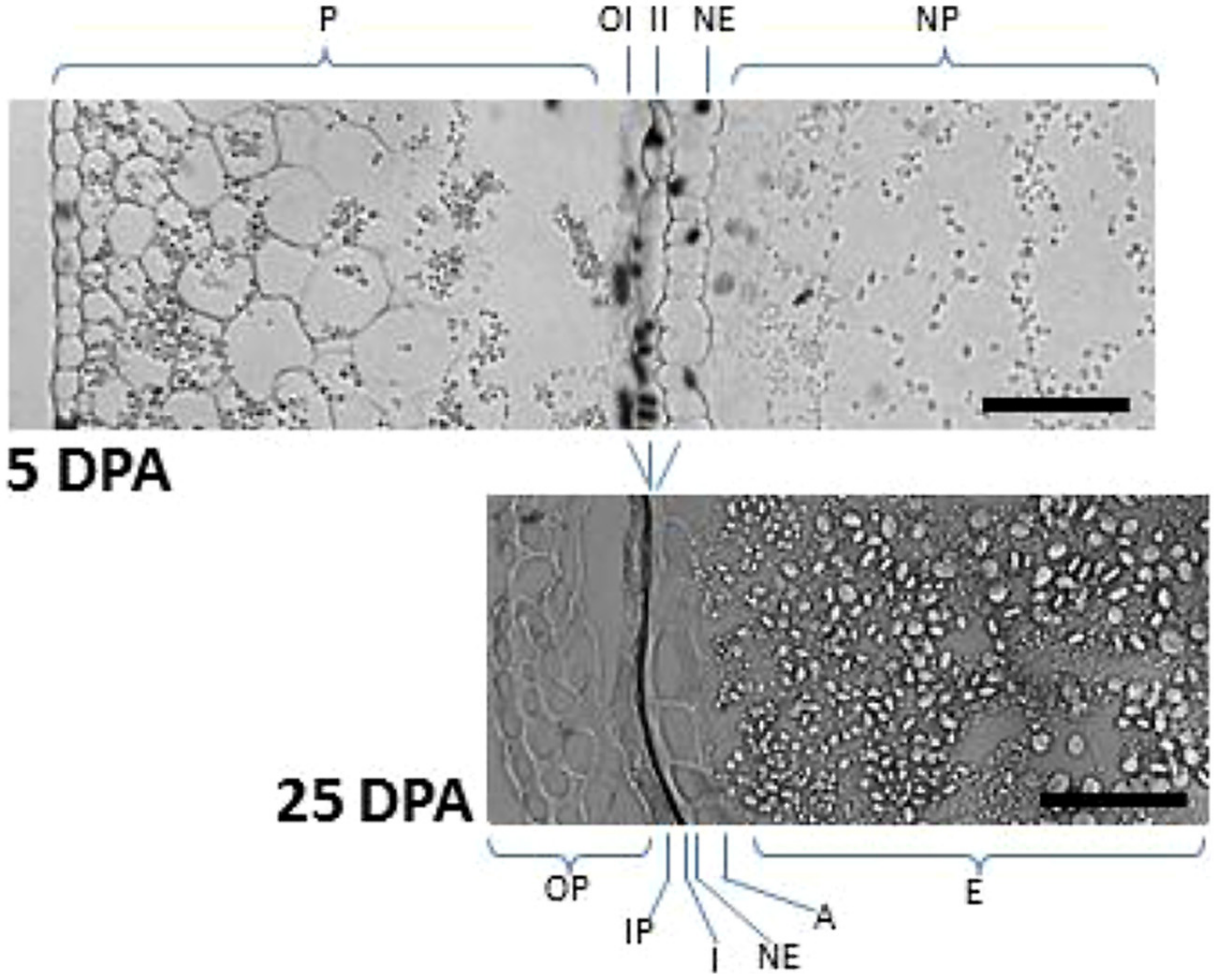
**TUNEL assay showing maternal tissues undergoing PCD at early and late stages of wheat seed development.** In seeds at 5 DPA, nuclei from the nucellus and the integuments showed TUNEL-positive signal. Note the reduction of pericarp width and the formation of seed cuticles as a consequence of PCD (compare 5 DPA vs 25 DPA). Starch granules of different sizes are also observed in the starchy endosperm. P, pericarp; OI, outer integument; II, inner integument; NE, nucellar epidermis; NP, nucellar parenchyma; OP, outer pericarp; IP, inner pericarp; I, pigmented cuticle derived from outer and inner integuments; A, aleurone; E, starchy endosperm. Bars, 50 μm.

At early stages of cereal seed development, the nucellus is among the first tissues to degenerate; nucellar cells undergoing a process of PCD, which has been well characterized at the morphological and biochemical levels (Domínguez et al., 2001; Radchuk et al., 2011). After the double fertilization event, the endosperm nucleus suffers several rounds of divisions to form a multinucleate syncytium surrounding the characteristic central vacuole (**Figure 2**). The technique of terminal deoxynucleotidil transferase dUTP end labeling (TUNEL), which allows the direct staining of fragmented DNA and, thus, the visualization of nuclei from cells undergoing PCD, has been of great aid in characterizing the pattern of PCD in early developing seeds. The TUNEL assay allowed the identification of degenerating nuclei of the inner cell layers of the nucellus very early after anthesis; the degenerative process spreading to the outer nucellar layers at 2 DPA (Radchuk et al., 2011). It has been proposed that PCD of the nucellus serves for the remobilization of its cellular contents, which are needed for the nourishment of the growing coenocyte and the cellularization process. Additional markers of cell degeneration are the expression of different hydrolytic enzymes, such as the aspartic protease nucellin (Chen and Foolad, 1997), a cathepsin B-like protease (Domínguez and Cejudo, 1998), the vacuolar processing enzyme nucellain (Linnestad et al., 1998), and the α-amylase AMY 4 (Radchuk et al., 2009). A gradient from internal to external layers is observed in the degeneration of the nucellus in developing wheat grains that culminates when only the nucellar epidermis remains. At 5 DPA, the nucellar parenchyma is completely disorganized, and TUNEL-labeled nuclei of the nucellar epidermis and the two-cell layer inner integuments are observed (**Figure 3**; Domínguez et al., 2001). At 15 DPA, the nucellus is reduced to a single-cell layer, which shows high level of expression of genes encoding cathepsin B-like thiol protease and serine carboxypeptidase III, suggesting a high hydrolytic activity in this tissue (Domínguez and Cejudo, 1998). It is noteworthy that besides that of genes involved in the hydrolytic activity, these nucellar cells also show the expression of genes encoding enzymes involved in biosynthetic metabolism. This is the case of phosphoenolpyruvate carboxylase (PEPC), which is expressed at high level at early stages of seed development (5 DPA) in the nucellus, the multinucleate syncytium and the vascular tissue. This activity may generate carbon skeletons to support the demand of amino acid biosynthesis in the growing endosperm (González et al., 1998).

### PERICARP

In the case of wheat and barley seeds, the pericarp is a tissue of maternal origin which, at early stages of development, is formed by several layers of parenchymatic cells, a two-cell-layer chlorenchyma and the inner epidermis (**Figure 2**). During the pre-storage phase (0–10 DPA), this tissue shows a decrease in cell divisions, which is accompanied by twofold to threefold increase in cell elongation, while the rows of parenchymatic cells localized between the inner integument and the outer epidermis are reduced twofold to fourfold (Radchuk et al., 2011). The first symptoms of pericarp cell degeneration appear at 4 DPA (Domínguez et al., 2001; Radchuk et al., 2011). Then, during the period of 6–10 DPA, PCD is extended to the whole tissue (Radchuk et al., 2011), so that by 15 DPA the pericarp is reduced to several layers of cuticle, as shown for the case of the wheat seed (**Figure 3**). Biochemical analyses showed the presence of proteolytic activities in the pericarp of wheat seeds at early stages of development (Domínguez and Cejudo, 1996). Furthermore, transcriptomic analyses performed in barley seeds revealed the expression of different genes encoding proteolytic enzymes in this tissue (Sreenivasulu et al., 2006), including the vacuolar processing enzyme VPE4 (Radchuk et al., 2011), suggesting that the degeneration of these cells occurs by a process of PCD. In addition, the expression of α-amylases suggests the existence of starch degradation in the pericarp (Radchuk et al., 2009). Indeed, pericarp PCD is accompanied by dynamic changes in starch accumulation patterns (**Figure 3**); starch granules being synthesized, deposited and degraded in plastids temporarily until their utilization by the growing endosperm (Zhou et al., 2009). PCD of the maternal tissues at these stages of seed development may have two purposes: to make room for the expanding endosperm and to support the nourishment of this tissue.

### NUCELLAR PROJECTIONS

The nucellar projections cells form a complex tissue of great relevance during seed development. These cells differentiate into transfer cells, thus enabling the transfer of nutrients from the pericarp to the endosperm cavity (**Figure 2**). Based on morphological features, several stages of differentiation through the radial axis can be established: roundish meristematic cells close to the pigment strand and adjacent to the vascular tissue (stage I); middle zone with elongated cells (stage II); transfer cells with peculiar cell wall invaginations (stage III); and autolysing cells adjacent to the endosperm cavity (stage IV). PCD plays a relevant function in this process of differentiation (Domínguez et al., 2001). While nucellar projection cells show no symptoms of PCD at early stages of development (5 DPA), most cells show TUNEL-stained nuclei as seed development progresses (13 DPA). At 18 DPA, TUNEL staining is restricted to nucellar projection cells adjacent to the pigment strand, because cells located near the endosperm cavity seem to be completely degraded (Domínguez et al., 2001). The high level of expression of cathepsin B-like thiol protease (Domínguez and Cejudo, 1998) and the vacuolar processing enzyme nucellain (Linnestad et al., 1998) in nucellar projection cells at 9–16 DPA is in agreement with the known role of these proteases in the process of cell degeneration. Another marker of the differentiation and PCD of the nucellar projections is the JEKILL protein (Radchuk et al., 2006). The increase in the level of this protein has been associated with structural changes in the nucellar projections, so that a gradient is generated from the crease region to the autolysing cells close to the endosperm cavity. Repression of the gene encoding JEKILL impairs the differentiation of the nucellar projections, which affects the exchange of nutrients between the pericarp and the endosperm (Radchuk et al., 2006). The gradient observed in the nucellar projections, from the crease region to the endosperm cavity, has also been observed by large-scale *in situ* hybridization expression analyses (Drea et al., 2005) and by laser micro-dissection pressure catapulting-based transcriptome analyses (Thiel et al., 2008). At 8 DPA, the meristematic zone (stage I of differentiation) shows the expression of genes characteristic of tissues with high mitotic activity. Genes involved in cell wall biosynthesis and expansin/extensin genes are expressed along the elongation zone (stage II); finally, genes involved in PCD-related proteolysis and nitrogen remobilization are expressed in the disintegration zone (stages III and IV). Several genes associated with the degeneration of the nucellar projection cells have been identified (Domínguez and Cejudo, 1998; Linnestad et al., 1998; Radchuk et al., 2006), however, little is known about upstream regulatory factors controlling their patterns of expression. The MADS29 transcription factor was proposed to play a relevant function in the control of nucellus and nucellar projection cells degeneration (Yin and Xue, 2012; Yang et al., 2012). The *MADS29* gene is expressed at high levels in the nucellus at early stages of seed development, up to 3 DPA. At later stages, 3–10 DPA, this gene shows a high level of expression in nucellar projection cells but not in the pericarp, integuments and endosperm (Yin and Xue, 2012). Under-expression of the *MADS29* gene provokes abnormal development and formation of shrunken seeds with reduced rate of grain-filling and altered starch granules. Histological analysis of these seeds revealed that the degeneration of the nucellar projection cells and other maternal tissues is blocked (Yang et al., 2012; Yin and Xue, 2012), suggesting that MADS29 directly regulates the expression of several PCD-related genes (Yin and Xue, 2012). Nutrient transport in temperate cereals, such as wheat and barley, occurs along the entire length of the grain through a single vascular band embedded in the maternal pericarp. This vascular band distributes nutrients to the endosperm cavity through the nucellar projection. However, in tropical crops, such as maize and sorghum, the transfer of nutrients occurs through a placento-chalazal layer localized in the basal region of the grain. Interestingly, like the nucellar projections, the placento-chalazal layer also degenerates during maize seed development (Kladnik et al., 2004).

## FILIAL TISSUES UNDERGO PCD IN DEVELOPING AND GERMINATING CEREAL SEEDS

With regard to the processes of PCD of filial tissues, these may be classified in those that occur during seed development, such as in the suspensor, embryo-surrounding layers and the starchy endosperm, and those taking place after germination, which include PCD in the parenchymal, epithelial, and vascular tissue of the scutellum, and the aleurone layer (**Figure 1**).

### SUSPENSOR AND EMBRYO-SURROUNDING LAYERS

In cereals such as maize, the first asymmetric division of the diploid zygote produces an apical cell, which develops into the embryo proper, and a basal cell, which generates the suspensor. The analysis of the pattern of expression of different genes suggests a marked regionalization in the differentiation of the embryo (Okamoto et al., 2005), which might also be affected by auxin transport (Forestan et al., 2010). Several tissues undergo PCD during embryogenesis. This is the case of the suspensor, which participates in the transfer of nutrients from maternal tissues to the developing embryo proper. The suspensor undergoes PCD in conjunction with PCD in the embryo-surrounding tissues. In maize seeds at 14 DPA, the scutellum cell layers that surround the shoot primordium, as well as the coleoptile and the root cap, show TUNEL-stained nuclei. Nuclei of the shoot primordium-surrounding layers appear completely degraded by 17 DPA (Giuliani et al., 2002). The suspensor undergoes a process of PCD, which is extended between 14 and 27 DPA defining a top-to-bottom gradient of DNA fragmentation, chromatin condensation and nuclei degeneration (Giuliani et al., 2002). In the so-called *emb* (embryo-specific) mutants of maize, which show arrested embryo development but a normal endosperm, the process of PCD in the suspensor is impaired (Consonni et al., 2003). More in-depth analyses at the molecular level have been carried out in non-cereal crops such as in the gymnosperm *Norway spruce* (Filonova et al., 2000). In this system it was identified a metacaspase, termed mcII-Pa, which has autoprocessing activity and is translocated from the cytoplasm to the nucleus in embryo cells undergoing PCD. Cell death thus relies on the proteolytic activity of metacaspase mcII-Pa, which acts as an executioner of PCD (Bozhkov et al., 2005). The death of the embryo suspensor requires the activation of autophagy-related components downstream of metacaspase mcII-Pa, as shown by the fact that the genetic suppression of the metacaspase-autophagy pathway promotes a switch from vacuolar PCD to necrosis. This suppression results in failure of suspensor differentiation and embryonic arrest (Minina et al., 2013). In addition, VEIDase, a caspase-6-like activity, was also identified in *Norway spruce* embryogenesis. The activity of this protease increases at early stages of embryo development and it has been proposed to participate in embryo pattern formation. When VEIDase activity is inhibited, the differentiation of the embryo-suspensor is blocked and the development of the embryo arrested (Bozhkov et al., 2004). VEIDase activity was also detected in barley developing embryo (Boren et al., 2006), suggesting common PCD mechanisms in gymnosperms and monocots. In tobacco, it was shown that the molecular mechanism triggering suspensor PCD is based on the antagonistic actions of two proteins: the cystatin NtCYS, a protease inhibitor, and its target, the cathepsin H-like protease NtCP14. NtCYS prevents precocious PCD in the basal cell of the proembryo by inhibiting NtCP14 protease. Transcriptional down-regulation of NtCYS leads to an increase in NtCP14 activity, which promotes PCD (Zhao et al., 2013). Silencing of the NtCYS inhibitor or overexpression of NtCP14 protease genes provoke precocious cell death with consequent embryonic arrest and grain abortion (Zhao et al., 2013).

### ENDOSPERM

In wheat seeds, the expansion of the endosperm is preceded by PCD in cells adjacent to the nucellar projections. The endosperm cavity is thus formed, allowing the transfer of nutrients from the vascular bundle embedded in the pericarp to the growing endosperm. The development of the endosperm in cereal seeds encompasses different processes, such as endoreduplication, accumulation of storage materials and PCD. During endoreduplication, DNA replication is not followed by cytokinesis, resulting in an altered cell cycle and polyploidy (Sabelli, 2012). In maize seeds, endoreduplication is initiated in the central area of the endosperm around 8–10 DPA; the process being extended toward the periphery and producing a high level of polyploidy in endosperm cells at 20 DPA (Sabelli, 2012). It should be noted that endoreduplication is not a homogeneous process since central endosperm cells have higher levels of polyploidy than external cells (Sabelli, 2012). The progression of PCD in the endosperm of developing maize seeds follows a two-wave pattern: the first wave begins around 16 DPA in central cells, coincident with an increase in DNA content; the second starts at the upper crown and progresses toward the base of the seed between 24 and 40 DPA, paralleling the pattern of starch accumulation (Young et al., 1997). In contrast with maize seeds, the pattern of PCD in the endosperm of developing wheat seeds progresses randomly; PCD being initiated by 16 DPA and extended in a random manner until 30 DPA, when the entire endosperm is affected (Young et al., 1997). Retinoblastoma-related (RBR) proteins and cyclin-dependent kinase (CDK) have been identified as fundamental players in cereal endosperm development. The retinoblastoma-related pathway seems to play a major role in endosperm development of maize seeds since it is involved in the control of processes such as endoreduplication, cell proliferation, cell size and cell death (Sabelli et al., 2013).

### EMBRYO VASCULAR TISSUE

In cereals, the differentiation of the scutellum vascular tissue proceeds only to the provascular stage of seed development, and is completed following germination. This pattern of differentiation has the purpose of avoiding translocation of nutrients from the scutellum to the embryonic axis before grain maturation (Swift and O’Brien, 1970, 1971). However, a fully functional vascular system is needed immediately after seed germination. Therefore, differentiation of the scutellar tracheary elements is completed in seeds at 2–3 days after imbibition (DAI), forming the characteristic annular thickenings, in a process that involves PCD (Domínguez et al., 2002). Differentiation of the tracheary elements implies the participation of endo- and exopeptidases. Up to six genes encoding carboxypeptidases have been identified in cereals (Dal Degan et al., 1994; Washio and Ishikawa, 1994); some of them being expressed in the embryo of germinating grains. Only the GA_3_-induced serine carboxypeptidase III, which was isolated from wheat aleurone cells (Baulcombe et al., 1987), has been shown to participate in vessel formation. The pattern of expression of this gene, as determined by in situ hybridization, shows a clear coincidence with TUNEL-stained nuclei in the tracheary elements of the scutellum, which suggests a role for this cartboxypeptidase as executioner in this PCD process (Domínguez et al., 2002). The participation of the serine carboxypeptidase III in the differentiation of vascular tissues of other organs, such as shoots and roots, has also been suggested (Domínguez et al., 2002).

### ALEURONE

The differentiation of the aleurone layer, the outermost cell layer of the endosperm, initiates in developing seeds around 8 DPA (Bosnes et al., 1992; **Figure 2**). In contrast with the endosperm, which undergoes a process of PCD at later stages of seed development (Young et al., 1997; Young and Gallie, 1999), the aleurone layer and the embryo remain alive in the mature seed. In wheat seeds, both tissues show the expression of protease inhibitors (Corre-Menguy et al., 2002), which may have a protective function since the surrounding pericarp shows intense proteolytic activity during seed development (Domínguez and Cejudo, 1996). Following germination, the aleurone layer displays a high metabolic activity to synthesize and secrete hydrolytic enzymes, which are released into the starchy endosperm and promote the degradation of storage reserves (Cejudo et al., 1992, 1995; Domínguez and Cejudo, 1995, 1999). Once this function is completed, the aleurone undergoes a process of PCD (Domínguez et al., 2004). DNA fragmentation in aleurone layer nuclei was observed in seeds after 4 DAI, and then increased progressively. TUNEL assays revealed a very characteristic spatial pattern of aleurone layer PCD; the process being initiated in cells proximal to the embryo and extended to distal cells, both in wheat and barley (Wang et al., 1998; Domínguez et al., 2004). In contrast to wheat and barley, the process of aleurone PCD was delayed in maize seeds; DNA laddering being detected only after 12 DAI (Domínguez et al., 2004). This delay may reflect differences in the germination strategies of both types of grains. In this regard, it should be noted that the scutellum is larger in maize than in wheat or barley seeds and, thus, may have a more relevant function supporting the initial stages of seedling growth. The progression of PCD in the aleurone layer is a tightly regulated process, which takes place only when these cells have completed the synthesis and secretion of hydrolytic enzymes. Indeed, this function of the aleurone cells is essential for germination as shown by the fact that the aleurone-deficient maize mutant seeds, *dek1*, are unable to germinate (Domínguez et al., 2004).

### SCUTELLUM

Although, as mentioned above, some of the tissues surrounding the embryo undergo PCD during embryogenesis in developing seeds, the bulk of scutellum PCD occurs after germination (Domínguez et al., 2012). This process was analyzed in wheat seeds in which the first symptoms of PCD appear at 4 DAI and increase progressively up to 7 DAI, affecting both the epidermal and parenchymal cells. The spatial progression of scutellum PCD in wheat grains occurs with an apical-to-basal gradient. PCD is initiated in the apical zone once the adjacent aleurone cells have completed PCD (Domínguez et al., 2012). This pattern of PCD progression suggests that one of the major functions of the scutellum in germinated seeds, which is the transfer of nutrients from the starchy endosperm to support the initial seedling growth (West et al., 1998; Aoki et al., 2006), does not cease abruptly (Domínguez et al., 2012). In fact, the degeneration of the scutellum seems to be coordinated with the sequential production of hydrolases. In wheat seeds at early stages of germination (1 DAI), the *AmyI* gene, which encodes the α-amylase I isoform, is expressed exclusively in the scutellar epithelium; its expression being transient and independent of GA_3_. At a later stage (2 DAI), *AmyI* expression in the scutellar epithelium decreases while it increases in the aleurone layer (Cejudo et al., 1995). In contrast, the gene encoding a cathepsin B-like is expressed in scutellum parenchymal cells but not in the epithelium of wheat seeds at 2 DAI (Cejudo et al., 1992), thus suggesting a function for this protease other than mobilization of the starchy endosperm. Whether cathepsin B-like is involved in a lysosomal-like function, in the final degradation of peptide products of the proteases secreted into the endosperm, or in pro-death roles, is not yet known. As mentioned above, the gene encoding the serine carboxipeptidase III is expressed during the differentiation of the scutellum vascular tissue (Domínguez et al., 2002). Therefore, PCD progression in scutellar parenchyma and epithelium appears as the last events in nutrients remobilization before the autonomous growth of the embryo (Domínguez et al., 2012).

## DIFFERENT HALLMARKS CHARACTERIZE THE PROCESS OF PCD IN CEREAL SEEDS

The well-defined patterns of PCD in developing and germinating cereal seeds, as well as the variety of cells undergoing PCD, has favored the use of these systems to study PCD at the biochemical level. One of the more characteristic hallmarks of PCD in most developmental processes is the fragmentation of DNA, which is highly dependent of the level of DNA packaging (Domínguez and Cejudo, 2012). Nuclear DNA is packed into chromatin loops of ca. 50 kb, six of which are grouped in a rosette-like structure. In cereal seeds at initial stages of endosperm development (4–6 DPA), the identification of DNA fragments of 50–300 kb suggests the participation of proteases that cleave chromatin folding points of the rosette-like structure as an early event in DNA fragmentation and PCD (Young and Gallie, 2000). The second stage of DNA degradation corresponds to the internucleosomal fragmentation, which results in the typical ladder of multimers of 180–200 bp. The analysis of endosperm PCD in developing cereal seeds identified internucleosomal laddering only at late stages of seed development (20 DPA to the end of maturation); this process being thus an irreversible phase in cell death (Young et al., 1997; Young and Gallie, 1999, 2000). Finally, a third stage of DNA fragmentation of the starchy endosperm cells occurs in germinated seeds, which yields completely digested nuclear DNA. Internucleosomal fragmentation of nuclear DNA, which is a hallmark of animal apoptosis, is a clear feature of PCD in cereal seeds as shown in maternal tissues (Domínguez et al., 2001; Domínguez and Cejudo, 2006) and starchy endosperm cells (Young et al., 1997; Young and Gallie, 1999, 2000) during development, as well as in aleurone layer (Wang et al., 1996; Domínguez et al., 2004) and epithelial and parenchymal cells of the scutellum (Domínguez et al., 2012) following germination.

The fact that DNA fragmentation is central to cell death implies the participation of nucleolytic enzymes in this process. Biochemical analyses of cells undergoing PCD in cereal seeds have allowed the identification of nuclear- and cytoplasmic-localized endonucleases. While nuclear-localized nucleases promote the cleavage of nuclear DNA into high- and low-molecular weight fragments, cytoplasmic-localized endonucleases participate in the degradation of naked, double- or single-stranded DNA fragments as the final step that culminates the complete degradation of the cellular DNA. The action of nuclear-localized endonucleases seems to be a key event in pre-mortem nuclear dismantling, whereas cytoplasmic-localized endonucleases seem to carry out the completion of DNA degradation after vacuolar tonoplast disruption during post-mortem nuclear dismantling (Domínguez and Cejudo, 2012). Wheat grains have been a model system to identify nuclear-localized factors involved in internucleosomal DNA fragmentation. Two nuclear-localized neutral Ca^2+^/Mg^2+^ endonucleases of ca. 30 and 50 kDa were identified, respectively, in aleurone (Domínguez et al., 2004) and nucellus cells undergoing PCD (Domínguez and Cejudo, 2006). An acid Zn^2+^-dependent endonuclease of ca. 70 kDa was also identified in the nucleus of wheat scutellum cells undergoing PCD (Domínguez et al., 2012). The differences in cation requirement, electrophoretic mobility and optimal pH reveal that internucleosomal DNA fragmentation is performed by different nucleases in the different tissues of the wheat grain (Domínguez et al., 2004, 2012; Domínguez and Cejudo, 2006). Among the so-called waste-management endonucleases responsible for a third level of DNA fragmentation, it is worth mentioning the nucleases acting in the starchy endosperm cells of germinating barley seeds (Brown and Ho, 1986, 1987).

Finally, *c*aspases are very well characterized proteases that participate as initiators and executioners in the process of apoptosis in animals. Because of this central role in the execution of apoptosis, the search for caspase counterparts has been a central focus of PCD studies in plants. Different approaches have revealed the complex set of endoproteolytic activities that participate in cell death in cereal seeds. These include serine-endoproteases in maternal tissues at early stages of development (Domínguez and Cejudo, 1996), and thiol-proteases in aleurone layer, scutellum, and starchy endosperm following germination (Domínguez and Cejudo, 1995). A caspase 6-like proteolytic activity, which acts at the sequence VEID, was identified in starchy endosperm and embryo cells from developing barley seeds (Boren et al., 2006). This VEIDase activity has been localized to autophagosome-like vesicles in randomly distributed cells of the starchy endosperm of barley seeds (Boren et al., 2006), in parallel with the progression of PCD in wheat (Young and Gallie, 1999). Therefore, VEIDase activity might be considered as an executioner with caspase-like activity in cereals. Nuclear-localized proteases have also been identified in cells undergoing PCD from developing wheat seeds. This is the case of a serine endoprotease of ca. 60 kDa identified in nuclear extracts from maternal tissues, which might be potentially responsible for the cleavage of structural proteins in the nucleus (Domínguez and Cejudo, 2006).

## HORMONAL REGULATION OF PCD IN DEVELOPING AND GERMINATING CEREAL SEEDS

**FIGURE 4:**
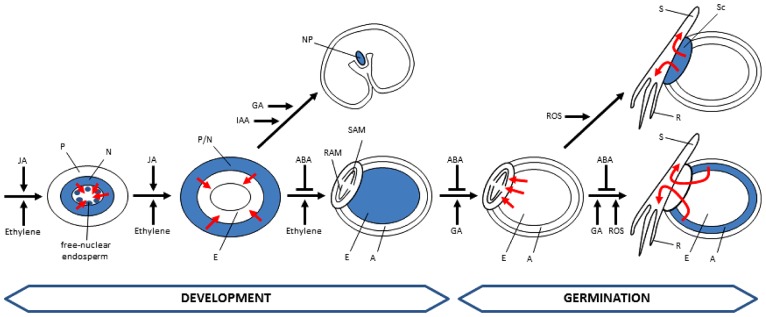
**Hormonal control of PCD in developing and germinating cereal seeds.** Tissues undergoing PCD are represented in blue. Fluxes of nutrients promoted by PCD events are indicated by red arrows. Cells of the starchy endosperm undergo PCD during development but remain intact until germination. A, aleurone; E, endosperm; N, nucellus; NP, nucellar projections; P, pericarp; R, root; RAM, root apical meristem; S, shoot; SAM, shoot apical meristem; Sc, scutellum; ABA, abscisic acid; IAA, indol acetic acid; JA, jasmonic acid; GA, gibberellic acid; ROS, reactive oxygen species.

The spatial-temporal patterns of PCD affecting different tissues of developing and germinating cereal grains, as described above, suggest the participation of mechanisms able to orchestrate these complex patterns of cell death. Although it is presumed that different factors are involved in the regulation of PCD in cereal seeds, hormonal action seems to be an obvious candidate. Yet the information concerning their participation in controlling the patterns of PCD in cereal seeds is scarce. Here, we summarize results showing the involvement of hormones in the control of PCD (**Figure 4**).

At early stages of *Arabidopsis* seed development, synergid cell death has been associated with the activation of the ethylene signaling pathway that takes place during the process of fertilization, in which EIN3 and EIN2 have been identified as critical factors (Völz et al., 2013). Ethylene appears also to be a crucial hormone controlling endosperm development in cereal seeds. During cereal seed development, two peaks of ethylene production occur; the first one being coincident with the onset of PCD in the central region of the endosperm, whereas the second one is associated with the increase in endonucleolytic activity (Young et al., 1997). Exogenously added ethylene results in the acceleration of cell death and DNA fragmentation in maize and wheat developing seeds, while treatments with inhibitors of ethylene biosynthesis or perception have the opposite effect (Young et al., 1997; Young and Gallie, 1999). The positive effect of ethylene on PCD induction was confirmed with the *shrunken2* mutant of maize, a starch-deficient mutant that accumulates sugars, thereby producing ethylene levels threefold to fivefold higher than in wild-type seeds, which shows accelerated PCD (Young et al., 1997).

Two hormones, jasmonic acid (JA) and ethylene, have been proposed to participate in the control of pericarp PCD, based on the high level of expression of genes involved in the biosynthesis and signaling of both hormones during PCD of maternal tissues (Sreenivasulu et al., 2006). Lipases, lipid transfer proteins and lipo-oxygenases, which are involved in biosynthesis of JA precursors, were found among the genes with increased expression in barley seeds at 6–12 DPA. In addition, genes of the ethylene signal transduction pathway are also induced at these stages of seed development. The genes showing a higher level of expression include the ethylene receptor (ETR3), the raf-like protein kinase (CTR1), a MAP kinase, the ethylene-insensitive (EIN2) and the ethylene response factor ERF2 (Sreenivasulu et al., 2006).

Gibberellins and auxins have also been proposed to participate in the control of PCD of maternal tissues, such as the nucellar projections. The gradient of differentiation observed in nucellar projection cells in wheat seeds at 8 DPA is coincident with the expression patterns of genes involved in GA biosynthesis and signaling (Thiel et al., 2008). In addition, the auxin-dependent MADS29 transcription factor appears as a key regulator of early seed development by stimulating the expression of a Cys-protease and other PCD-related proteins participating in the degradation of the nucellus and the nucellar projections (Yin and Xue, 2012).

The important function of ABA in the control of cereal seed maturation is well known. During seed development, ABA biosynthesis takes place both in the developing embryo and the endosperm with the participation of different members of the aldehyde oxidase gene family (Sreenivasulu et al., 2006). Furthermore, ABA plays an essential role in the acquisition of desiccation tolerance of the embryo by stimulating stress-responsive genes, such as those encoding late-embryogenesis abundant proteins and dehydrins (Sreenivasulu et al., 2006). The implication of ABA in cereal endosperm PCD was demonstrated with two maize *viviparous (vp)* mutants, the ABA-insensitive *vp1* and the ABA-deficient *vp9* mutants. It should be noted that ethylene levels in the developing endosperm of these mutants are twofold to fourfold higher than in wild-type seeds, and thus ethylene might promote an acceleration of cell death in these mutants. The treatment of wild-type seeds with fluridone, an inhibitor of ABA biosynthesis, also promoted DNA fragmentation and cell death. Based on these findings, ABA was proposed as a negative regulator of ethylene biosynthesis and/or action during maize development (Young and Gallie, 2000). Thus a balance between ABA and ethylene regulates the onset and progression of PCD during cereal endosperm development (Young et al., 1997; Young and Gallie, 1999, 2000).

While ABA has a key function in seed maturation by inhibiting precocious germination, gibberellins have the opposite function and are the most relevant hormones promoting seed germination. The aleurone layer, the only endosperm tissue that remains alive in mature cereal seeds, shows a high sensitivity to gibberellins. The response of aleurone cells to gibberellins may be subdivided in two phases. In the short term, gibberellins stimulate the metabolic activation of these quiescent cells upon seed imbibition. In response to gibberellins, aleurone cells induce the expression of genes encoding hydrolytic enzymes, such as amylases and proteases, which are secreted into the starchy endosperm (Cejudo et al., 1992, 1995; Domínguez and Cejudo, 1995, 1999). In addition, the hormone triggers the acidification of the starchy endosperm, thus facilitating the mobilization of storage compounds (Domínguez and Cejudo, 1999). Once the metabolic activation is achieved, aleurone cells show a long-term response to gibberellins, which involves the induction of PCD, a process that is counteracted by ABA (Wang et al., 1996, 1998; Bethke et al., 1999; Domínguez et al., 2004). Treatments of wheat grains with paclobutrazol, an inhibitor of gibberellin synthesis, delayed germination and avoided DNA fragmentation (Domínguez et al., 2004). Similarly, aleurone protoplasts treated with LY83583, which has an antagonistic effect on gibberellin signaling, showed no symptoms of PCD (Bethke et al., 1999). The activating role of gibberellins in PCD was corroborated with the analysis of the wheat gibberellin-insensitive mutant *Rht,* which shows altered post-germination, including delayed aleurone PCD (Domínguez et al., 2004). Taken into account the spatial–temporal gradients observed in the wheat aleurone layer, which affect the expression of genes encoding hydrolytic enzymes (Cejudo et al., 1992, 1995; Domínguez and Cejudo, 1999), the acidification of the starchy endosperm (Domínguez and Cejudo, 1999), and the process of DNA fragmentation and cell death (Domínguez et al., 2004), a model was generated to explain the action of gibberellins during the different PCD phases of a single aleurone cell: (1) quiescent phase, which is the status of aleurone cells in mature grains prior to gibberellin perception; (2) active phase, in which gibberellin perception induces the synthesis of hydrolytic enzymes and the acidification of the starchy endosperm; and (3) death phase, in which aleurone cells undergo PCD (Cejudo et al., 2001). According to this model, the aleurone layer of germinating grains is composed by a heterogeneous group of cells: those located adjacent to the embryo are the first to perceive and respond to gibberellins and, thus, to enter the death phase, whereas those located far from the embryo may be still in the quiescent phase (Cejudo et al., 2001).

## REDOX REGULATION OF PCD IN DEVELOPING AND GERMINATING CEREAL SEEDS

In addition to hormone control, redox regulation has been considered to play an important role in establishing the patterns of PCD in cereal seeds. Aerobic metabolism inevitably generates reactive oxygen species (ROS), including hydrogen peroxide, superoxide anion and hydroxyl radicals. These compounds show reactivity with almost all cellular macromolecules and, hence, their accumulation above certain levels may have a harmful effect causing oxidative stress (Bailly, 2004). At the end of the phase of development, the cereal seed enters a phase of desiccation, which involves a massive loss of water that provokes oxidative stress in tissues that remain alive in the mature seed, such as the embryo and the aleurone layer. Moreover, these tissues also suffer oxidative stress in germinating seeds due to resumption of respiration (Serrato and Cejudo, 2003). ROS, in conjunction with hormones, has been proposed to exert an important regulatory role in the regulation of PCD of cereal aleurone cells (Bethke and Jones, 2001; Fath et al., 2002). The GA-induced progression of PCD in these cells is accelerated in the presence of internally generated or exogenously applied hydrogen peroxide, whereas antioxidant agents, such as ascorbic acid or dithiothreitol, have the opposite effect (Bethke and Jones, 2001). Nitric oxide (NO) has the effect of delaying PCD of aleurone cells, most probably by counteracting the accelerating effect of ROS (Beligni et al., 2002). To avoid the harmful effect of ROS and prevent precocious PCD, aleurone cells are equipped with detoxification systems based on the scavenger activity of catalase, ascorbate peroxidase and superoxide dismutase (Fath et al., 2001), as well as haem oxygenase (Wu et al., 2011), some of which are down-regulated before the process of PCD is initiated (Fath et al., 2001). A nuclear detoxification system has also been described in wheat seed cells suffering oxidative stress, which is composed by an NADPH thioredoxin reductase (NTR) and a 1-Cys-peroxiredoxin (1-Cys Prx; Stacy et al., 1996, 1999; Pulido et al., 2009). This nuclear-localized redox system may have the function of preventing oxidative damage to DNA and nuclear structures, as suggested by DNA protection assays *in vitro*. In addition, this redox system may control the level of hydrogen peroxide in the nucleus, thus having a potential signaling function. Besides nuclear-localized detoxification systems, wheat aleurone cells also display a set of gibberellin-induced glycosylases participating in the base excision repair pathway to remove non-bulky DNA base lesions generated by ROS (Bissenbaev et al., 2011). Under the oxidant conditions generated in germinating seeds, the 1-Cys Prx undergoes a progressive inactivation by overoxidation of its catalytic cysteine residue (Pulido et al., 2009). This inactivation would promote a further accumulation of hydrogen peroxide; the nuclear environment becoming more oxidant and promoting cell death.

## CONCLUDING REMARKS AND PERSPECTIVES

The cereal seed constitutes an excellent example illustrating the essential function of cell death for developmental programs in plants. Different techniques, such as TUNEL staining of nuclei of dying cell, have been a valuable aid in identifying tissues undergoing PCD and also in studying the spatial-temporal patterns of PCD in developing and germinating cereal seeds. Moreover, although most of the tissues undergoing PCD in cereal seeds present autophagic-like morphology, other tissues such as the starchy endosperm show peculiar characteristics. The identification of proteolytic and nucleolytic activities associated with PCD in the different tissues reveal the biochemical complexity of cell death in this plant model system. PCD in cereal seeds occurs with very well-defined spatial-temporal patterns, which are established by different factors; hormones playing a relevant function. So far, most of the knowledge of PCD in cereal seeds has been obtained from descriptive approaches. However, the last decade has brought impressive advance in our understanding of the cereal genomes that have been sequenced. In cereal models such as rice and *Brachypodium*, larger collections of mutants are available; in addition, genetic transformation of cereals is becoming a routine technology. All these molecular and genetic tools now available for cereals are expected to facilitate functional approaches applied to the study of PCD, which may allow a more precise dissection of the process in this plant system and, eventually, the identification of genes acting as key regulators of cell death in plants.

## Conflict of Interest Statement

The authors declare that the research was conducted in the absence of any commercial or financial relationships that could be construed as a potential conflict of interest.
